# Impaired sensitivity to thyroid hormones is associated with hyperuricemia in a Chinese euthyroid population

**DOI:** 10.3389/fendo.2023.1132543

**Published:** 2023-04-19

**Authors:** Yingning Lu, Jie Wang, Yu An, Jia Liu, Ying Wang, Guang Wang, Song Leng

**Affiliations:** ^1^ Health Management Center, The Second Hospital of Dalian Medical University, Dalian, China; ^2^ Department of Endocrinology, Beijing Chao-Yang Hospital, Capital Medical University, Beijing, China

**Keywords:** sensitivity to thyroid hormones, uric acid, euthyroid, cardiovascular disease risk, Chinese population

## Abstract

**Objective:**

Impaired sensitivity to thyroid hormones has been reported as a common metabolic disorder, and it remains poorly understood whether it interplays with uric acid (UA) metabolism as an established risk factor for cardiovascular diseases (CVDs). We aimed to investigate the relationship between thyroid hormone sensitivity and elevated UA in a Chinese euthyroid population.

**Methods:**

A total of 15,955 euthyroid subjects were included in this study. Thyroid hormone sensitivity indices were calculated, including the thyroid feedback quantile-based index (TFQI), the Chinese-referenced parametric TFQI (PTFQI), the TSH index (TSHI), and the thyrotropin thyroxine resistance index (TT4RI), and the FT3/FT4 ratio. Linear and logistic regression analyses were performed to detect the association between thyroid hormone sensitivity and elevated UA.

**Results:**

Subjects with reduced sensitivity to thyroid hormones had increased UA levels in both genders (*p* for trend < 0.001). Logistic and linear regression analyses showed that higher TFQI, PTFQI, TSHI, and TT4RI were positively associated with elevated UA levels, but negatively associated with the FT3/FT4 ratio. The odds ratio (OR) of the highest versus the first quartile of TFQI was 1.20 (1.05, 1.38) in men and 1.80 (1.46, 2.23) in women (*p* < 0.001). PTHQI, TSHI, and TT4RI obtained similar results in both genders. Conversely, the highest quartile of the FT3/FT4 ratio was negatively correlated with elevated UA levels [men: OR 0.78 (0.68,0.89), women: OR 0.66 (0.53,0.81)].

**Conclusion:**

Impaired sensitivity to thyroid hormones was associated with elevated UA levels in euthyroid subjects. Our findings shed light on the role of thyroid hormone sensitivity in UA metabolism.

## Introduction

Thyroid hormones are key regulators of energy metabolism, and a close association with metabolic disorders has been demonstrated, even when thyroid hormones are within the normal range (1). Most patients with subclinical hypothyroidism (SCH) who are characterized by the combination of normal thyroid hormones and raised thyroid-stimulating hormone (TSH) are asymptomatic. Emerging evidence claimed that thyroid function was associated with uric acid (UA) metabolism (2). Hyperuricemia (HUA) is not only a precursor of gout and kidney failure, but also an important risk factor, contributing to the development of metabolic syndrome (MetS) and cardiovascular diseases (CVDs) (3–5). Both epidemiologic and genetic research support the essential element of HUA in the CVD progression (5).

However, data regarding the associations of TSH and thyroid hormones with UA metabolism are inconsistent. Some studies reported a positive relationship between thyroid function and HUA in both overt hypothyroidism (OH) and SCH (2, 6). This close association between thyroid function and UA metabolism has also been reported in the euthyroid population (2). Men with elevated TSH have a 1.89-fold risk for HUA compared to women, suggesting a gender-related regulation of TSH levels on UA metabolism. Conversely, a large study of 2,359 patients with thyroid dysfunction found no significant association between TSH and HUA. Moreover, another cross-sectional study revealed that only the impact of elevated free thyroxine (FT4) on UA metabolism is significant in a general population without OH (7). Therefore, the potential underlying mechanism of the conflicting association between thyroid function and UA metabolism remains unclear.

Physiologically, thyroid hormones and TSH inversely interact with each other because of a negative feedback loop of the hypothalamic–pituitary–thyroid (HPT) axis (8). However, the common co-occurrence of high TSH and thyroid hormones levels seems not to be reasonable, due to the negative physiological feedback loop. Recently, Laclaustra et al. hypothesized a possible acquired thyroid hormone resistance, which may be enough to explain these controversial results (8). In the past few years, most studies investigated the association of thyroid function with metabolic disorders by using a single index. Instead of a single index, the composite index takes an advantage of comprehensively reflecting the dynamic variability between thyroid hormones and TSH in a certain population. Thus, Laclaustra et al. proposed the thyroid feedback quantile index (TFQI), focusing on the deviations of the average pituitary responses to thyroid hormones in a general population, and linking it with metabolic disorders. TFQI has been used for accurately evaluating the central sensitivity to thyroid hormones, and other composite indices reflecting the central sensitivity to thyroid hormones include the thyroxine resistance index (TT4RI), the TSH index (TSHI), and the parametric thyroid feedback quantile-based index (PTFQI) (8). PTHQI was developed to quantify the estimated pituitary inhibition by thyroid hormones in the general population, which ranges from −1 to +1, with negative values indicating good sensitivity to FT4 and positive values, the opposite. The peripheral variability of thyroid hormones can be evaluated by the free thyroxine (FT3)/FT4 ratio. Previous research showed that the risk of HUA was increased in patients with SCH (2), and L-T4 replacement therapy could decrease serum UA levels in patients with recent onset SCH, indicating the influential role of thyroid hormones for modulating UA metabolism (9). However, in euthyroid subjects, the association of thyroid hormone sensitivity with HUA remains unclear. Therefore, it is of great significance for the Chinese general population to explore the effect of thyroid hormone sensitivity on UA metabolism. In this study, we aimed to gain insight into the interaction and sex-specific relationship between thyroid hormone sensitivity and HUA in the Chinese euthyroid population.

## Methods

### Study population

**Figure 1:**
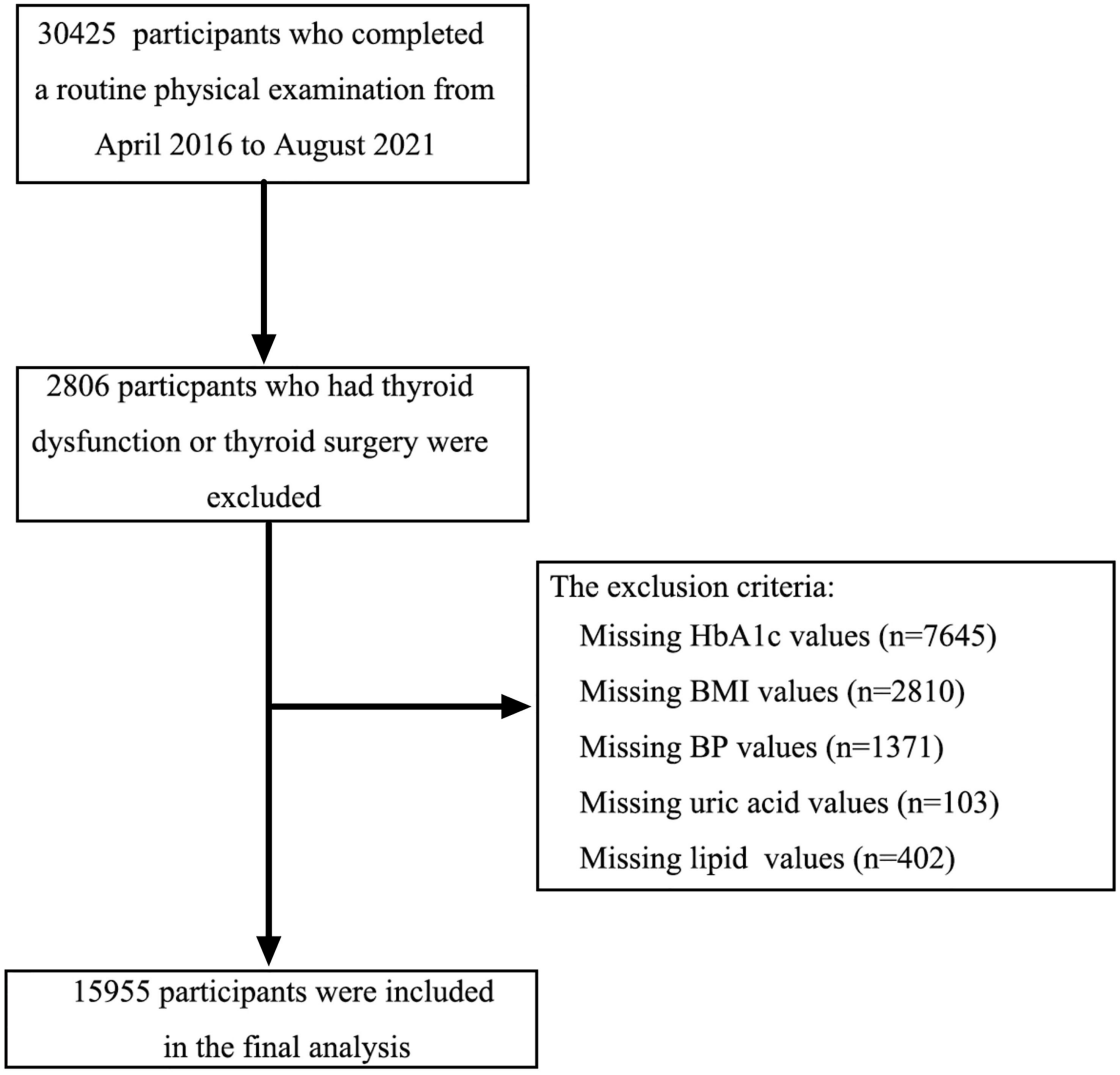
The flowchart of the study population.

This cross-sectional study enrolled participants (aged ≥ 18 years) who underwent a routine physical examination from April 2016 to August 2021 in Beijing Chao-yang Hospital. The inclusion criteria were as follows: (1) TSH between 0.55 and 4.78 μIU/ml); (2) FT4 between 11.45 and 22.65 pmol/L; (3) FT3 between 3.54 and 6.47 pmol/L; (4) subjects who were not pregnant; (5) subjects not taking iodine-containing drugs or contrast agents in the past 3 months; (6) subjects without a history of thyroid diseases and antithyroid therapy; (7) subjects without a history of hormone replacement treatment; and (8) subjects without a history of severe hepatic, renal dysfunction and cancer. Finally, 15,955 subjects were enrolled in this analysis (**Figure 1**). All participants provided written informed consent, and the study protocol was approved by the Ethics Committee of Beijing Chao-yang Hospital.

### Data collection and definitions

Blood samples were collected in the morning after overnight fasting at least 8 h, and body weight, height, and blood pressure were measured according to standard protocols by the same trained staff as previously described (10). TSH, FT4, and free triiodothyronine (FT3) were evaluated by electrochemiluminescence immunoassay using an Abbott Architect i2000 (Abbott Diagnostics, Abbott Park, IL, USA) as previously described (11). Serum triglyceride (TG), total cholesterol (TC), high-density lipoprotein cholesterol (HDL-C), low-density lipoprotein cholesterol (LDL-C), fasting blood glucose (FBG) colorimetric enzymatic assays using a biochemical auto-analyzer, UA, and hemoglobin A1c (HbA1c) were measured as previously described (12).

Euthyroidism was defined as having normal thyroid function according to FT3 (3.54–6.47 pmol/L), FT4 (11.45–22.65 pmol/L), and TSH (0.55-4.78 μIU/ml) within the reference ranges. HUA was defined as serum UA level ≥420 μmol/L in men and ≥360 μmol/L in women (13). Body mass index (BMI) was calculated as weight divided by height squared (kg/m ^2^). Diabetes was defined as FBG levels ≥ 7.0 mmol/L, currently treated with insulin or oral hypoglycemic agents, or a self-reported history (14). Hypertension was defined as systolic blood pressure (SBP) ≥ 140 mmHg or diastolic blood pressure (DBP) ≥ 90 mmHg, currently taking antihypertensive agents. Dyslipidemia was defined as TC ≥ 5.2 mmol/L or LDL-C ≥ 3.4 mmol/L or HDL-C < 1.0 mmol/L or TG ≥ 1.7 mmol/L as previously described (15). Fatty liver was diagnosed by three well-trained and experienced clinicians by hepatic ultrasound.

### Indices of thyroid hormone sensitivity

The calculation of thyroid hormone sensitivity includes central and peripheral indices of thyroid hormone sensitivity. Four different indices were calculated to evaluate central sensitivity to thyroid hormones, namely, TFQI, TSHI, TT4RI, and PTHQI (8). TFQI was calculated as cumulative distribution function (cdf) FT4 − (1 − cdf TSH) (8). TSHI was calculated as Ln TSH (μIU/ml) + 0.1345 × FT4 (pmol/L) (16). TT4RI was calculated as FT4 (pmol/L) × TSH (μIU/ml) (17). The value of TFQI is between −1 and 1. Positive values indicate decreased thyroid hormone sensitivity and negative values indicate increased thyroid hormone sensitivity. TFQI value is 0, indicating normal thyroid hormone sensitivity. The higher TSHI and TT4RI could indicate the poorer central sensitivity to thyroid hormones. For TSHI and TT4RI, higher values indicated lower central sensitivity to thyroid hormones. In addition, to improve the applicability of this research, a new index (PTHQI) was also calculated, which can be computed for any new value and adapted to any population from FT4 in pmol/L and TSH in mU/L using the standard normal cumulative distribution function as follows: Φ ((FT4 − μFT4)/σFT4) − (1 – Φ ((ln TSH − μln TSH)/σln TSH)), where μFT4 = 16.3802, σFT4 = 1.98049, μln TSH = 0.5865, and σln TSH = 0.43854 for the Chinese population.

The peripheral index of thyroid hormone sensitivity was calculated as follows: FT3/FT4 ratio = FT3 (pmol/L)/FT4 (pmol/L). The higher the FT3/FT4 ratio, the better peripheral sensitivity to thyroid hormones.

### Statistical analysis

All analyses were performed using Empower(R) (www.empowerstats.com, X&Y Solutions Inc., Boston, MA) and R (http://www.Rproject.org). The odds ratios (ORs) and corresponding 95% confidence intervals (95% CIs) were calculated. All statistical tests were two-sided, and *p*-values < 0.05 were considered statistically significant.

The characteristics of subjects were presented as means ± standard deviations (SDs) for continuous variables with a normal distribution, and median (Q1–Q3) for continuous variables with a non-normal distribution. The normality of the variables was assessed using the Kolmogorov–Smirnov test. Continuous variables with a non-normal distribution were compared using Kruskal–Wallis test, and those with a normal distribution were compared using Student’s *t*-tests. Categorical variables were expressed as numbers (%) and the chi-square test was applied for the comparison of groups.

Sex-specific linear regression analysis was performed to explore the association between serum UA levels and indices of thyroid hormone sensitivity. The results were expressed as standardized coefficient *β* and *p*-value. Model 1 was unadjusted. Model 2 was adjusted for age and BMI. Model 3 was adjusted for age, BMI, hypertension, diabetes, dyslipidemia, and fatty liver.

The general linear model with one-way analysis of covariance (ANCOVA) was applied to compare UA levels across indices of thyroid hormone sensitivity by adjusting for age and BMI. *p* for trend was calculated by linear regression analyses. Multivariable logistic regression analysis was conducted to evaluate the associations between UA and thyroid hormone sensitivity using three models after adjusting for potential confounding factors. Model 1 was unadjusted for age and BMI. Model 2 was further adjusted for diabetes based on Model 2. Model 3 was further adjusted for hypertension, dyslipidemia, and fatty liver based on Model 2.

## Results

### Characteristics of study population by hyperuricemia

Clinical characteristics of men and women with and without HUA are shown in **Table 1**. This cross-sectional study included 8,179 men and 7,776 women. The median UA levels were 359.00 (322.00–389.00) μmol/L in men without HUA and 470.00 (443.25–513.00) μmol/L in men with HUA. The median UA levels were 273.00 (240.00–306.00) μmol/L in women without HUA and 391.00 (373.00–420.00) μmol/L in women with HUA. In the subjects with HUA, women tended to be older; had lower BMI, SBP, DBP, FBG, TG, LDL-C, UA, FT3, FT4, TFQI, PTHQI, and FT3/FT4 ratio; had higher TC, HDL-C, TSH, and TT4RI; and were less likely to be hypertensive, diabetic, dyslipidemia, or fatty liver patients compared with men with HUA. In the subjects without HUA, women tended to be younger; had lower BMI, SBP, DBP, FBG, HBA1c, TG, TC, LDL-C, UA, FT3, FT4, TFQI, TSHI, PTHQI, and FT3/FT4 ratio; had higher HDL-C, TSH, and TT4RI; and were less likely to be hypertensive, diabetic, dyslipidemia, or fatty liver patients compared with men without HUA. However, it is obvious that men and women have different reference ranges, with women having a lower reference range, which may lead to these results. Of note, the FT3, FT4, and TSH levels were higher in the subjects with HUA compared with those without HUA in both genders (all *p* < 0.001).

**Table 1:** Clinical characteristics of men and women with and without HUA.

Characteristics	Non-hyperuricemia		*p*-value	Hyperuricemia		*p*-value
	Men	Women		Men	Women	
*N*	5,201	6,888		2,978	888	
Age, years	44.30 (13.15)	40.27 (12.60)	<0.001	41.20 (12.05)	44.21 (14.91)	<0.001
BMI, kg/m^2^	25.09 (3.22)	22.50 (3.37)	<0.001	26.76 (3.60)	24.81 (4.25)	<0.001
SBP, mm/Hg	124.98 (15.81)	115.40 (16.56)	<0.001	126.83 (15.66)	121.51 (19.06)	<0.001
DBP, mm/Hg	74.45 (11.07)	67.57 (10.40)	<0.001	76.38 (11.67)	71.02 (11.48)	<0.001
FBG, mmol/L	4.91 (4.55–5.34)	4.73 (4.44–5.05)	<0.001	4.93 (4.58–5.38)	4.88 (4.51–5.35)	0.097
HBA1c, %	5.50 (5.20–5.70)	5.40 (5.20–5.60)	<0.001	5.50 (5.30–5.70)	5.50 (5.30–5.90)	0.003
TG, mmol/L	1.30 (0.95–1.84)	0.93 (0.71–1.29)	<0.001	1.70 (1.18–2.44)	1.35 (0.93–1.99)	<0.001
TC, mmol/L	4.81 (4.27–5.40)	4.73 (4.22–5.35)	0.072	5.00 (4.46–5.62)	5.07 (4.42–5.69)	0.181
HDL-C, mmol/L	1.20 (1.00–1.40)	1.50 (1.30–1.74)	<0.001	1.10 (1.00–1.30)	1.30 (1.10–1.50)	<0.001
LDL-C, mmol/L	3.00 (2.47–3.50)	2.70 (2.20–3.25)	<0.001	3.11 (2.60–3.70)	3.06 (2.50–3.70)	0.141
UA, μmol/L	359.00 (322.00–389.00)	273.00 (240.00–306.00)	<0.001	470.00 (443.25–513.00)	391.00 (373.00–420.00)	<0.001
FT3, pmol/L	5.14 (0.47)	4.67 (0.45)	<0.001	5.19 (0.48)	4.71 (0.46)	<0.001
FT4, pmol/L	16.54 (1.99)	15.51 (1.83)	<0.001	16.73 (2.02)	15.76 (1.89)	<0.001
TSH, pmol/L	1.90 (0.83)	2.16 (0.93)	<0.001	1.97 (0.86)	2.26 (0.99)	<0.001
TFQI	−0.04 (−0.22–0.14)	−0.15 (−0.31–0.03)	<0.001	−0.01 (−0.20–0.17)	−0.11 (−0.27–0.08)	<0.001
TT4RI	28.61 (20.93–38.78)	30.66 (22.23–41.98)	<0.001	30.24 (22.04–40.50)	32.61 (22.92–45.22)	<0.001
TSHI	2.78 (2.43–3.11)	2.77 (2.43–3.10)	0.095	2.84 (2.50–3.17)	2.84 (2.50–3.19)	0.728
PTHQI	−0.00 (−0.28–0.27)	−0.06 (−0.34–0.18)	<0.001	0.04 (−0.23–0.33)	−0.01 (−0.28–0.24)	<0.001
FT3/FT4 ratio	0.31 (0.29–0.34)	0.30 (0.28–0.33)	<0.001	0.31 (0.29–0.34)	0.30 (0.27–0.33)	<0.001
Hypertension, *n* (%)	964 (18.53%)	643 (9.34%)	<0.001	630 (21.16%)	168 (18.92%)	0.148
Diabetes, *n* (%)	340 (6.54%)	133 (1.93%)	<0.001	128 (4.30%)	28 (3.15%)	0.128
Dyslipidemia, *n* (%)	3,068 (58.99%)	2,698 (39.17%)	<0.001	2,246 (75.42%)	547 (61.60%)	<0.001
Fatty liver, *n* (%)	1,852 (35.61%)	946 (13.73%)	<0.001	1,760 (59.10%)	388 (43.69%)	<0.001

Data are expressed as the mean ± SD or median (Q1–Q3) or number (%). Bold indicates p-value < 0.05.

BMI, body mass index; SBP, systolic blood pressure; DBP, diastolic blood pressure; FBG, fasting blood glucose; HbA1c, glycated hemoglobin; TG, triglycerides; TC, total cholesterol; HDL-C, high-density lipoprotein cholesterol; LDL-C, low-density lipoprotein cholesterol; UA, uric acid; HUA, hyperuricemia; FT3, free triiodothyronine; FT4, free thyroxine; TSH, thyrotropin; TFQI, thyroid feedback quantile-based index; PTFQI, parametric thyroid feedback quantile-based index; TSHI, TSH index; TT4RI, thyrotropin thyroxine resistance index.

### Association between thyroid hormone sensitivity and serum UA levels

**Figure 2:**
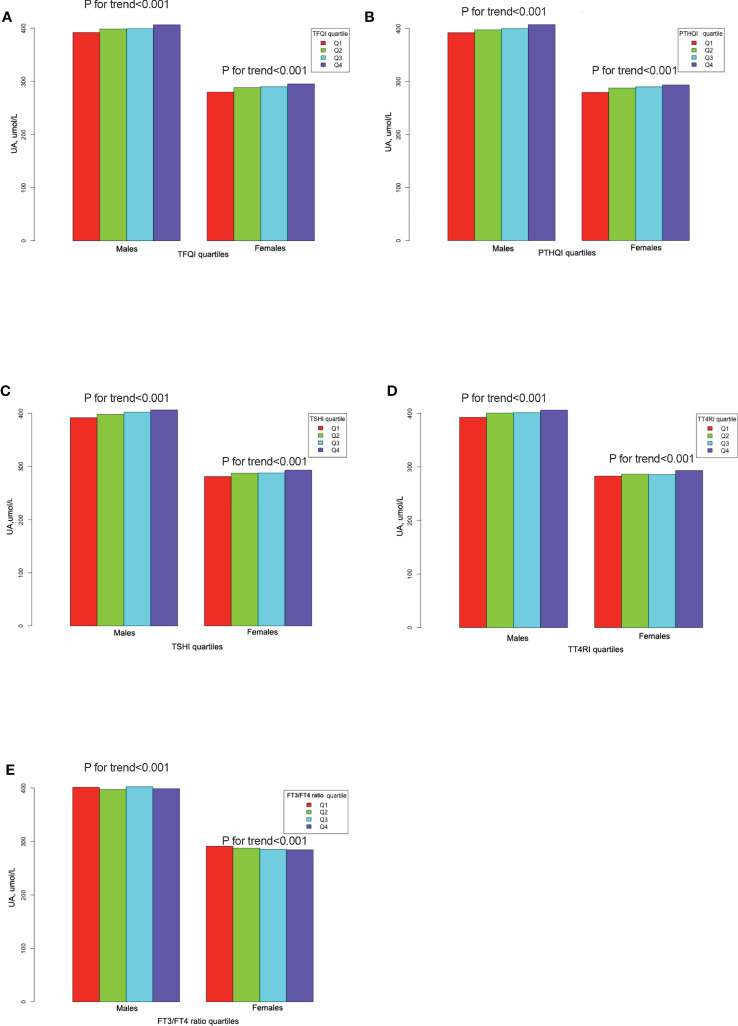
The UA levels across thyroid parameter quartiles. **(A)** The UA levels across TFQI quartiles. **(B)** The UA levels across PTFQI quartiles. **(C)** The UA levels across TSHI quartiles. **(D)** The UA levels across TT4RI quartiles. **(E)** The UA levels across FT3/FT4 ratio quartiles.

Serum UA levels increased linearly from the lowest to the highest quartiles of TFQI, TT4RI, TSHI, and PTFQI in both genders, but decreased from the lowest to the highest quartiles of the FT3/FT4 ratio (all *p* for trend <0.001) (**Figure 2**). As shown in **Table 2**, linear regression analysis showed that serum UA levels increased progressively with increased TFQI, PTFQI, TSHI, and TT4RI in both genders, even after adjusting for confounding factors (all *p* < 0.05). Conversely, FT3/FT4 ratio levels exhibited a negative association with UA levels in both genders (all *p* < 0.001). Moreover, the associations of FT4 and TSH with UA levels were more obvious in women than in men.

**Table 2:** Linear regression analysis for the association between thyroid parameters and UA levels in a euthyroid population.

Variables	Model 1	Model 2	Model 3
	*β* (95% CI) *p*-value	*β* (95% CI) *p*-value	*β* (95% CI) *p*-value
Men			
FT3	0.151 (0.102, 0.200) <0.001	−0.025 (−0.073, 0.024) 0.320	−0.066 (−0.114, −0.019) 0.006
FT4	0.099 (0.061, 0.137) <0.001	0.072 (0.036, 0.108) <0.001	0.057 (0.022, 0.093) 0.002
TSH	0.021 (0.011, 0.032) <0.001	0.012 (0.002, 0.022) 0.016	0.010 (0.001, 0.020) 0.035
TFQI	0.058 (0.040, 0.077) <0.001	0.037 (0.019, 0.054) <0.001	0.033 (0.016, 0.050) <0.001
TT4RI	0.028 (0.018, 0.038) <0.001	0.017 (0.007, 0.027) <0.001	0.014 (0.005, 0.024) 0.003
TSHI	0.078 (0.053, 0.102) <0.001	0.049 (0.025, 0.073) <0.001	0.040 (0.017, 0.063) <0.001
PTHQI	0.037 (0.026, 0.049) <0.001	0.022 (0.011, 0.033) <0.001	0.020 (0.009, 0.030) <0.001
FT3/FT4 ratio	−0.008 (−0.043, 0.027) 0.646	−0.072 (−0.105, −0.039) <0.001	−0.079 (−0.112, −0.047) <0.001
Women			
FT3	0.068 (0.018, 0.118) 0.008	0.055 (0.006, 0.103) 0.028	0.027 (−0.021, 0.074) 0.266
FT4	0.149 (0.108, 0.190) <0.001	0.203 (0.164, 0.243) <0.001	0.182 (0.143, 0.221) <0.001
TSH	0.019 (0.008, 0.030) <0.001	0.014 (0.004, 0.024) 0.008	0.012 (0.002, 0.023) 0.016
TFQI	0.085 (0.066, 0.105) <0.001	0.101 (0.082, 0.120) <0.001	0.091 (0.073, 0.110) <0.001
TT4RI	0.029 (0.018, 0.039) <0.001	0.027 (0.017, 0.038) <0.001	0.024 (0.014, 0.034) <0.001
TSHI	0.088 (0.063, 0.114) <0.001	0.093 (0.068, 0.118) <0.001	0.083 (0.059, 0.107) <0.001
PTHQI	0.050 (0.038, 0.063) <0.001	0.055 (0.043, 0.068) <0.001	0.050 (0.038, 0.062) <0.001
FT3/FT4 ratio	−0.087 (−0.125, −0.050) <0.001	−0.141 (−0.177, −0.104) <0.001	−0.138 (−0.174, −0.102) <0.001

Data are expressed as standardized coefficients (β) and 95% CI. Continuous variables with a non-normal distribution (UA, FT3, FT4, TSH, TFQI, TT4RI, TSHI, PTHQI, and FT3/FT4 ratio) were log transformed for analysis.

Model 1: Crude model.

Model 2: Adjusted for age and BMI.

Model 3: Adjusted for age, BMI, diabetes, dyslipidemia, fatty liver, and hypertension.

### Association between sensitivity to thyroid hormone indices and hyperuricemia by gender

Given the differences in serum UA levels between men and women, we further explore the relationship between sensitivity to thyroid hormone indices and HUA, and logistic regression analyses were conducted in both genders (**Table 3**). The increased TFQI, TT4RI, TSHI, and PTHQI were positively associated with HUA in both genders. Conversely, the FT3/FT4 ratio was negatively associated with HUA. In men, each 1 SD increase of TFQI (*β* = 1.38, 95% CI: 1.14, 1.68, *p* = 0.001), TT4RI (*β* = 1.19, 95% CI: 1.08, 1.31, *p* < 0.001), TSHI (*β* = 1.01, 95% CI: 1.00, 1.01, *p* = 0.002), and PTHQI (*β* = 1.22, 95% CI: 1.08, 1.38, *p* = 0.002) was positively associated with HUA in men, even after further adjusting for various potential confounders, whereas each 1 SD increase of the FT3/FT4 ratio (*β* = 0.11, 95% CI: 0.03, 0.37, *p* < 0.001) was negatively associated with HUA (**Table 3**). To be noted, the associations between sensitivity to thyroid hormones and HUA were more obvious in women than in men in **Table 3**. In women, each 1 SD increase of TFQI (*β* = 2.40, 95% CI: 1.78, 2.33, *p* < 0.001), TT4RI (*β* = 1.35, 95% CI: 1.17, 1.57, *p* < 0.001), TSHI (*β* = 1.01, 95% CI: 1.00, 1.01, *p* < 0.001), and PTHQI (*β* = 1.62, 95% CI: 1.34, 1.97, *p* < 0.001) was positively associated with HUA, while each 1 SD increase of the FT3/FT4 ratio (*β* = 0.03, 95% CI: 0.01, 0.21, *p* < 0.001) was negatively associated with HUA.

**Table 3:** Logistical regression analysis for the association between thyroid hormone sensitivity and HUA in a euthyroid population.

Variables	Model 1	Model 2	Model 3
	*β* (95% CI) *p*-value	*β* (95% CI) *p*-value	*β* (95% CI) *p*-value
Men			
TFQI	1.61 (1.34, 1.93) <0.001	1.43 (1.18, 1.73) <0.001	1.38 (1.14, 1.68) 0.001
TT4RI	1.29 (1.18, 1.42) <0.001	1.21 (1.10, 1.33) <0.001	1.19 (1.08, 1.31) <0.001
TSHI	1.01 (1.00, 1.01) <0.001	1.01 (1.00, 1.01) <0.001	1.01 (1.00, 1.01) 0.002
PTHQI	1.36 (1.21, 1.52) <0.001	1.24 (1.10, 1.40) <0.001	1.22 (1.08, 1.38) 0.002
FT3/FT4 ratio	0.81 (0.27, 2.42) 0.700	0.19 (0.06, 0.60) 0.005	0.11 (0.03, 0.37) <0.001
Women			
TFQI	2.06 (1.55, 2.72) <0.001	2.54 (1.90, 3.39) <0.001	2.40 (1.78, 3.23) <0.001
TT4RI	1.34 (1.16, 1.55) <0.001	1.38 (1.19, 1.60) <0.001	1.35 (1.17, 1.57) <0.001
TSHI	1.01 (1.00, 1.01) <0.001	1.01 (1.00, 1.01) <0.001	1.01 (1.00, 1.01) <0.001
PTHQI	1.53 (1.28, 1.84) <0.001	1.67 (1.38, 2.02) <0.001	1.62 (1.34, 1.97) <0.001
FT3/FT4 ratio	0.27 (0.04, 1.65) 0.157	0.04 (0.01, 0.28) 0.001	0.03 (0.01, 0.21) <0.001

Model 1: Crude model.

Model 2: Adjusted for age and BMI.

Model 3: Adjusted for age, BMI, diabetes, dyslipidemia, fatty liver, and hypertension.

**Figure 3:**
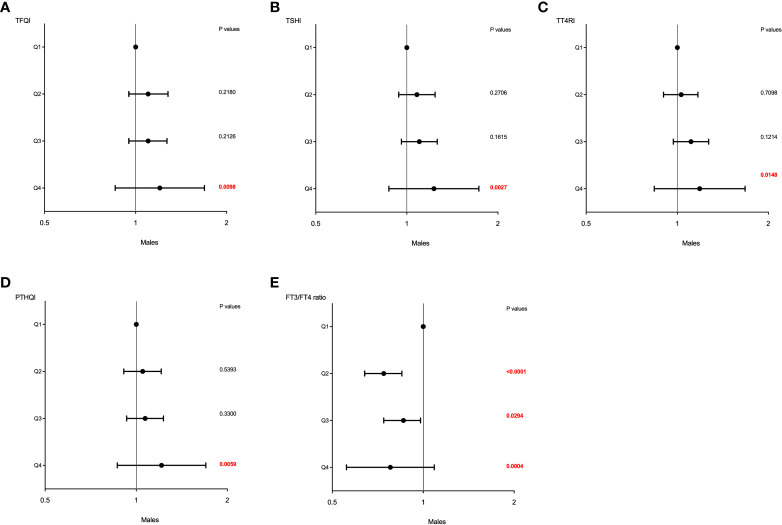
The forest maps of logistic regression analysis for the association between HUA and thyroid hormone sensitivity in men. **(A)** The ORs for HUA across TFQI quartiles. **(B)** The ORs for HUA across TSHI quartiles. **(C)** The ORs for HUA across TT4RI quartiles. **(D)** The ORs for HUA across PTHQI quartiles. **(E)** The ORs for HUA across FT3/FT4 ratio quartiles. Model was adjusted for age, BMI, dyslipidemia, diabetes, fatty liver, and hypertension. Q1, the first quartile, Q2, the second quartile, Q3, the third quartile, Q4, the fourth quartile.

**Figure 4:**
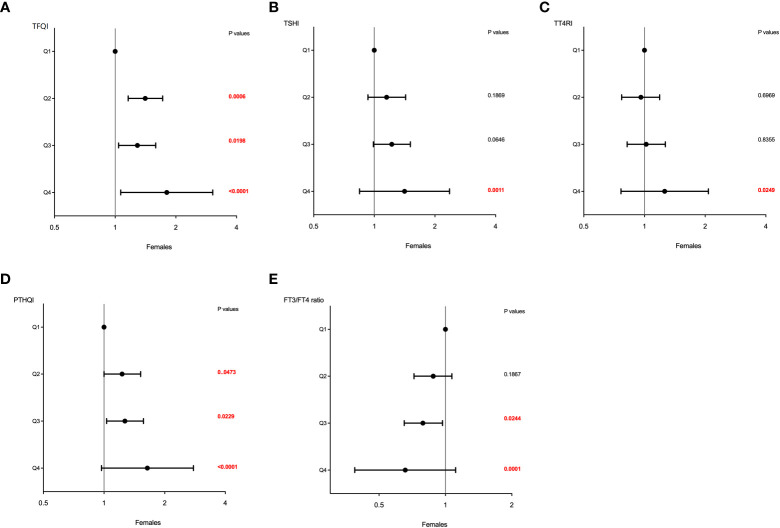
The forest maps of logistic regression analysis for the association between HUA and thyroid hormone sensitivity in women. **(A)** The ORs for HUA across TFQI quartiles. **(B)** The ORs for HUA across TSHI quartiles. **(C)** The ORs for HUA across TT4RI quartiles. **(D)** The ORs for HUA across PTHQI quartiles. **(E)** The ORs for HUA across FT3/FT4 ratio quartiles. Model was adjusted for age, BMI, dyslipidemia, diabetes, fatty liver, and hypertension. Q1, the first quartile, Q2, the second quartile, Q3, the third quartile, Q4, the fourth quartile.

Similar effects of sex on the association between sensitivity to thyroid hormone index quartiles and HUA were also observed and shown in **Table 4**. The higher quartiles of TFQI, TT4RI, TSHI, and PTHQI showed a significant positive association with HUA, whereas FT3/TT4 was negatively associated with HUA in both genders. It was also worth noting that these associations remained significant even after adjusting for confounding factors as shown in **Table 4** and **Figure 3**. In men, the ORs of Q3 and Q4 versus the Q1 TFQI for having HUA showed increasing trends after adjusting for age, BMI, diabetes, dyslipidemia, fatty liver, and hypertension (Q3: OR = 1.10, 95% CI: 0.95, 1.27, Q4: OR = 1.20, 95% CI: 1.05, 1.38, *p* for trend =0.014). Consistently, the ORs of Q2, Q3, and Q4 versus the Q1 TT4RI were 1.03 (95% CI: 0.90, 1.17), 1.11 (95% CI: 0.97, 1.27), and 1.19 (95% CI: 1.03,1.36), respectively (*p* for trend = 0.007). Similar results of TSHI and PTHQI quartiles were obtained (TSHI: Q2: OR = 1.08, 95% CI: 0.94, 1.24, Q3: OR = 1.10, 95% CI: 0.96, 1.26, Q4: OR = 1.23, 95% CI: 1.07, 1.41, *p* for trend = 0.003; PTHQI: Q2: OR = 1.05, 95% CI: 0.91, 1.21, Q3: OR = 1.07, 95% CI: 0.93, 1.23, Q4: OR = 1.21, 95% CI: 1.06, 1.39, *p* for trend = 0.005). By contrast, there was a negative association between FT3/FT4 ratio quartiles and HUA in men (Q2: OR = 0.74, 95% CI: 0.64, 0.85, Q3: OR = 0.86, 95% CI: 0.74, 0.98, Q4: OR = 0.78, 95% CI: 0.68, 0.89, *p* for trend = 0.012). Accordingly, the associations were more apparent in women, as shown in **Table 4** and **Figure 4**. The ORs of Q2, Q3, and Q4 versus the Q1 TFQI were 1.41 (95% CI: 1.16, 1.72), 1.29 (95% CI: 1.04, 1.59), and 1.80 (95% CI: 1.46, 2.23), respectively (*p* for trend <0.001). Similarly, the higher TT4RI, TSHI, and PTHI quartiles were significantly related with elevated UA levels in women, as shown in **Table 4**. Conversely, the higher FT3/FT4 ratio quartiles showed the lower odds for having elevated UA levels (Q2: OR = 0.88, 95% CI: 0.72, 1.07, Q3: OR = 0.79, 95% CI: 0.65 0.97, Q4: OR = 0.66, 95% CI: 0.53, 0.81, *p* for trend <0.001). These results indicated the potential effects of sex hormones on the relationship between thyroid hormone sensitivity and elevated UA levels in the euthyroid population.

**Table 4:** Logistical regression analysis for the association between thyroid hormone sensitivity quartiles and HUA in a euthyroid population.

Variables	Model 1	Model 2	Model 3
	*β* (95% CI)	*β* (95% CI)	*β* (95% CI)
Men			
TFQI			
Q1	1.0	1.0	1.0
Q2	1.13 (0.98, 1.30)	1.11 (0.96, 1.28)	1.10 (0.95, 1.28)
Q3	1.15 (1.01, 1.32)	1.11 (0.96, 1.28)	1.10 (0.95, 1.27)
Q4	1.32 (1.16, 1.50)	1.23 (1.07, 1.41)	1.20 (1.05, 1.38)
*P* for trend	<0.001	0.005	0.014
TT4RI			
Q1	1.0	1.0	1.0
Q2	1.12 (0.98, 1.27)	1.06 (0.93, 1.21)	1.03 (0.90, 1.17)
Q3	1.20 (1.06, 1.36)	1.12 (0.98, 1.28)	1.11 (0.97, 1.27)
Q4	1.32 (1.16, 1.50)	1.22 (1.07, 1.39)	1.19 (1.03, 1.36)
*P* for trend	<0.001	0.004	0.007
TSHI			
Q1	1.0	1.0	1.0
Q2	1.13 (0.99, 1.29)	1.10 (0.96, 1.26)	1.08 (0.94, 1.24)
Q3	1.20 (1.06, 1.37)	1.12 (0.98, 1.28)	1.10 (0.96, 1.26)
Q4	1.36 (1.19, 1.54)	1.25 (1.10, 1.43)	1.23 (1.07, 1.41)
*P* for trend	<0.001	0.001	0.003
PTHQI			
Q1	1.0	1.0	1.0
Q2	1.08 (0.94, 1.23)	1.06 (0.93, 1.22)	1.05 (0.91, 1.21)
Q3	1.16 (1.01, 1.32)	1.09 (0.95, 1.25)	1.07 (0.93, 1.23)
Q4	1.35 (1.18, 1.53)	1.24 (1.09, 1.42)	1.21 (1.06, 1.39)
*P* for trend	<0.001	0.002	0.005
FT3/FT4 ratio			
Q1	1.0	1.0	1.0
Q2	0.81 (0.71, 0.93)	0.75 (0.65, 0.87)	0.74 (0.64, 0.85)
Q3	0.98 (0.86, 1.12)	0.89 (0.78, 1.03)	0.86 (0.74, 0.98)
Q4	0.96 (0.84, 1.09)	0.82 (0.71, 0.93)	0.78 (0.68, 0.89)
*P* for trend	0.631	0.066	0.012
Women			
TFQI			
Q1	1.0	1.0	1.0
Q2	1.35 (1.12, 1.63)	1.40 (1.15, 1.70)	1.41 (1.16, 1.72)
Q3	1.19 (0.97, 1.46)	1.31 (1.06, 1.61)	1.29 (1.04, 1.59)
Q4	1.63 (1.33, 1.99)	1.86 (1.51, 2.29)	1.80 (1.46, 2.23)
*P* for trend	<0.001	<0.001	<0.001
TT4RI			
Q1	1.0	1.0	1.0
Q2	0.99 (0.80, 1.22)	0.97 (0.78, 1.20)	0.96 (0.77, 1.19)
Q3	1.03 (0.84, 1.27)	1.02 (0.82, 1.26)	1.02 (0.82, 1.27)
Q4	1.32 (1.09, 1.61)	1.28 (1.05, 1.56)	1.26 (1.03, 1.54)
*P* for trend	0.003	0.007	0.013
TSHI			
Q1	1.0	1.0	1.0
Q2	1.18 (0.96, 1.45)	1.19 (0.97, 1.47)	1.15 (0.93, 1.43)
Q3	1.21 (0.99, 1.49)	1.21 (0.99, 1.50)	1.22 (0.99, 1.51)
Q4	1.44 (1.18, 1.75)	1.48 (1.20, 1.81)	1.41 (1.15, 1.74)
*P* for trend	<0.001	<0.001	<0.001
PTHQI			
Q1	1.0	1.0	1.0
Q2	1.23 (1.01, 1.50)	1.22 (1.00, 1.50)	1.23 (1.00, 1.52)
Q3	1.28 (1.05, 1.56)	1.26 (1.03, 1.55)	1.27 (1.03, 1.57)
Q4	1.54 (1.26, 1.89)	1.68 (1.36, 2.06)	1.64 (1.33, 2.03)
*P* for trend	<0.001	<0.001	<0.001
FT3/FT4 ratio			
Q1	1.0	1.0	1.0
Q2	0.94 (0.78, 1.13)	0.88 (0.73, 1.06)	0.88 (0.72, 1.07)
Q3	0.90 (0.74, 1.09)	0.80 (0.66, 0.97)	0.79 (0.65, 0.97)
Q4	0.84 (0.69, 1.03)	0.69 (0.56, 0.85)	0.66 (0.53, 0.81)
*P* for trend	0.084	<0.001	<0.001

Model 1: Crude model.

Model 2: Adjusted for age and BMI.

Model 3: Adjusted for age, BMI, diabetes, dyslipidemia, fatty liver, and hypertension.

## Discussion

In this study, we found that both impaired central and peripheral sensitivity to thyroid hormones (increased TFQI, TT4RI, TSHI, and PTHQI, and decreased FT3/FT4 ratio) were associated with elevated UA levels in a Chinese euthyroid population. In addition, we observed that the associations of thyroid hormone sensitivity indices with elevated UA levels were more apparent in women than in men, suggesting the potential impact of sex-related thyroid hormone sensitivity on UA metabolism.

UA is the final product of purine metabolism, which is produced by xanthine oxidase (XOR) (18, 19). Serum UA is generated from the degradation of nucleic acids, including DNA and RNA, or muscle breakdown, and is affected by age and high-salt and glucose-rich diets. HUA is a metabolic disease characterized by an abnormally high serum UA concentration, mainly induced by UA overproduction or underexcretion (20). HUA has been shown to be one of the most important metabolic agents by the findings of studies conducted in recent years. Some studies demonstrated that HUA is associated not only with gout but also with numerous cardiometabolic diseases, such as hypertension, MetS, diabetes, and obesity (21). Previous research established that elevated serum UA is a risk factor for these diseases (22, 23). The association between HUA and hypertension is independent of conventional cardiovascular risk factors (24), and two previous meta-analyses concluded that HUA was well predictive of incident hypertension (25, 26). Epidemiological and genetic studies confirmed a positive relationship between elevated serum UA levels and MetS, and the effect of sex on this association has been validated in other several studies (27, 28). Every 1 mg/dl increase in serum UA conferred approximately 1.17-fold increased risk for MetS in young women. The risk is much higher than age-matched men and older women (1.17 *vs*. 1.05 and 1.04, respectively, *p* < 0.05) (28). Many studies underlined increased serum UA as an important pathogenic factor for not only gout but also CVD, including myocardial infarction, stroke, and heart failure (29, 30). Several pathophysiologic mechanisms may be responsible for the relationship between elevated serum UA levels and cardiometabolic diseases, including the imbalance of pro-oxidant and anti-oxidant effects, vascular endothelial function, inflammation, and the activation of the renin–angiotensin–aldosterone system (RAAS) (31–33). Additionally, the UA-dependent mitochondrial oxidative stress can drive the inhibition of fatty acid oxidation and stimulation of lipogenesis, finally inducing the development of fatty liver (34). Therefore, findings from our study such as the higher prevalence of hypertension, diabetes, dyslipidemia, and fatty liver in HUA can be explained. Several lines of evidence suggested that increased serum UA levels are always accompanied by aging and male sex, consistent with our findings. Notably, we also observed a positive association between central sensitivity to thyroid hormones (TFQI, TT4RI, TSHI, and PTHQI) and elevated serum UA levels, and a negative association of the FT3/FT4 ratio. These results indicated a close association of impaired central and peripheral sensitivity to thyroid hormones with HUA in euthyroid subjects.

The existing results of the relationship between thyroid function and urate metabolism were inconsistent. Some earlier studies reported no significant association between TSH and UA in hyperthyroid and euthyroid patients (35), while others opposed it, demonstrating that elevated TSH increased the risk of HUA (2). Two previous studies proved a high incidence of HUA in hypothyroid and hyperthyroid subjects (36, 37). Moreover, in subjects with normal thyroid function, FT4 levels have shown to be positively and linearly associated with serum UA, and consequently, the prevalence of HUA was elevated with increasing FT4 levels (7, 38). On the other hand, in patients with recent onset SCH, L-T4 replacement therapy could decrease serum UA levels by improving insulin sensitivity, indicating the influential role of thyroid hormones for modulating UA metabolism (9). These conflicting results would seem to be not fully explained by metabolic mechanisms described in clinical thyroid diseases, including the different responsiveness of different tissues to thyroid hormones, the hypothalamic–pituitary–thyroid feedback loop, and an inherited syndrome with co-occurrence of high TSH and FT3 or FT4. Nevertheless, the regulation of UA metabolism in euthyroid subjects remains unclear.

Recently, Laclaustra et al. suggested that reduced sensitivity to thyroid hormone could justify the conflicting findings previously reported and not just the existence of RTH (8). Reduced sensitivity to thyroid hormone is a more common disorder in the general population than RTH. Therefore, new indices TFQI and PTHQI have been proposed by Laclaustra et al. to evaluate central sensitivity to thyroid hormone. It has been reported that higher TFQI and PTHQI were associated with obesity, MetS, and diabetes in that study, indicating the effect of impaired central sensitivity to thyroid hormone on metabolism. Besides the close correlation between TFQI and cardiometabolic health characteristics, TFQI has been found superior to PTHQI, TT4RI, and TSHI for reflecting central sensitivity to thyroid hormone. Another study illustrated that the new index TFQI was significantly associated with diabetes and hypertension in euthyroid subjects (39). Also, PTHQI has been reported to be associated with metabolic diseases and CVD (40). Consistent with these results, we found a positive association with the prevalence of HTN, diabetes, and dyslipidemia. Moreover, a recent cross-sectional study from China showed that the odds of HUA were increased in subclinical hypothyroid subjects with high TFQI. In line with that, we yielded similar conclusions in a euthyroid population, suggesting a potential interaction between thyroid hormone resistance and UA metabolism. Notably, these studies only focused on TFQI, but not FT4 or TSH levels within normal range, which was different from our results. In our study, not only FT4, but also TSH values were observed to be significantly higher in the HUA group of both genders, hinting at a central thyroid hormone resistance state. Accordingly, a negative association between the FT3/FT4 ratio and elevated UA was found in both genders, indicating the impact of peripheral sensitivity to thyroid hormone.

Given the joint effect of thyroid hormones, composite indices, standing for thyroid homeostasis, were calculated to estimate the responsiveness to thyroid system in this current study. In a euthyroid population, both impaired central and peripheral sensitivity to thyroid hormone ratio were associated with elevated UA. A more stable and apparent association of TFQI with UA was observed compared to TSHI and TT4RI, especially in women. TSHI and TT4RI showed a weaker association than TFQI, possibly derived by TSH and FT4 extreme values. Both TFQI and PTHQI incorporated FT4 and TSH in calculating thyroid hormone resistance, presenting the joint distribution of FT4 and TSH values with the advantage of not yielding thyroid hormone parameter extreme values. TT4RI and TSHI were representative of extreme values of FT4 and TSH, based on the formula. Consequently, the associations observed for TT4RI and TSHI are prone to align with the existing relationship between FT4 or TSH and UA, respectively. Notably, we observed a stronger association between TFQI and UA metabolism in women, which can possibly be explained by women having much higher sex hormone binding globulin (SHBG) and lower testosterone levels compared with men. Another possibility is the gene-by-sex interaction, because both hypothyroidism and hyperthyroidism are more prevalent in women than in men (41). We emphasized that resistance to thyroid hormones is a common disorder in euthyroid subjects, and TFQI is a new index for measuring central sensitivity to the grade of pituitary gland inhibition by FT4. Our study shed light on the potential interaction of reduced sensitivity to thyroid hormones with UA metabolism and subsequent risk of CVD. Importantly, early recognition of resistance to thyroid hormones, under euthyroidism conditions, may be a prognostic factor in HAU subjects in the clinical practice.

The current study has some limitations. Firstly, being a cross-sectional study, the causality between reduced sensitivity to thyroid hormones and elevated UA cannot be established. Secondly, we did not collect data of eating habits and thyroid-related antibodies, which might affect serum UA levels. Thirdly, because the participants underwent a routine physical examination at the medical health center, the data about sex hormones were not collected. The strong association between symptoms of thyroid changes and menopausal symptoms, which may be somewhat overlapping, should be noted. Finally, because the data of this research were from a Chinese population, it is uncertain whether our findings can be applicable to other ethnic backgrounds. More prospective studies are necessary to validate the causal association between impaired sensitivity to thyroid hormones and elevated UA.

In conclusion, we found that both impaired central and peripheral sensitivity to thyroid hormones were associated with elevated UA in a Chinese euthyroid population. Our study provided new insights into the impact of thyroid hormone sensitivity on serum UA metabolism and lay the groundwork for future therapeutic strategies for UA-related CVD risk. Further studies are needed to explore the potential mechanism.

## Data availability statement

The datasets presented in this article are not readily available because some or all datasets generated during and/or analyzed during the current study are not publicly available but are available from the corresponding author on reasonable request. Requests to access the datasets should be directed to Song Leng. Ph.D E-mail: dllengsong@126.com.

## Ethics statement

The studies involving human participants were reviewed and approved by All participants provided written informed consent, and the study protocol was approved by the Ethics Committee of Beijing Chao-yang Hospital. The patients/participants provided their written informed consent to participate in this study.

## Author contributions

All authors contributed to the study conception and design. YL and JW performed formal analysis and wrote the manuscript. Study design, material preparation, and data collection were performed by YL, JW, YA, JL, GW, YW and SL. The paper was revised by SL. All authors contributed to the article and approved the submitted version.
